# IgE, anti-IgE therapy, and regulatory T cells: new paradigms in allergic inflammation

**DOI:** 10.3389/falgy.2026.1759921

**Published:** 2026-02-04

**Authors:** C. Benito-Villalvilla, A. Angelina, L. Martín-Cruz, S. Sirvent, O. Palomares

**Affiliations:** 1 Department of Biochemistry and Molecular Biology, School of Chemistry, Complutense University, Madrid, Spain; 2 Department of Biochemistry and Molecular Biology, School of Medicine, Complutense University, Madrid, Spain; 3 Department of Biochemistry and Molecular Biology, School of Pharmacy, Complutense University, Madrid, Spain

**Keywords:** allergic inflammation, anti-IgE, dendritic cells, IgE, regulatory T cells

## Abstract

Allergic inflammation arises from a dysregulated immune response in which immunoglobulin E (IgE) plays a central pathogenic role. By binding to its high-affinity FcεRI on mast cells and basophils, IgE orchestrates the rapid activation and mediator release that underlies immediate hypersensitivity reactions. Compelling experimental evidence indicates that the pathogenic role of IgE extends well beyond of the mere effector cells degranulation, contributing also to shape antigen presentation, to regulate the function of dendritic cells, and to endorse an immune environment that favours type 2 inflammation. Within this complex network, regulatory T cells (Tregs) serve as a critical counterbalance, maintaining tolerance to environmental allergens and restraining excessive type 2 inflammatory responses. Individuals with allergic diseases often display quantitative and/or functional alterations within the Tregs compartment, including signs of phenotypic plasticity shifted toward pro-allergic states. These observations raise important questions about how IgE-mediated signalling pathways might directly or indirectly impair proper Tregs development or stability. In this context, anti-IgE therapies such as omalizumab have shown that, beyond reducing free IgE levels and downregulating FcεRI expression, they may also promote the expansion or restoration of Tregs, which might well contribute to the reestablishment of immune tolerance. Deciphering the interplay among IgE, Tregs, and anti-IgE agents can help to pave the way towards the development of innovative disease-modifying strategies for allergic diseases and other inflammatory immune-mediated diseases.

## Introduction

The rising incidence of allergy over the last decades has positioned allergic diseases as a major global health burden (1, 2). Asthma, allergic rhinitis, food and drug allergies, or atopic dermatitis significantly impairs the quality of life of millions of people worldwide both children and adults, imposing considerable social and economic challenges. The development of these conditions arises from multifactorial mechanisms involving genetic predisposition, environmental exposures, and lifestyle changes (2). Allergic diseases are characterized by aberrant type 2 inflammatory responses driven by complex interactions among epithelial cells, the innate and adaptive immune systems, and environmental allergens and exposome.

The allergic immunological cascade begins when allergens contact and disrupt epithelial barrier surfaces, triggering the release of alarmins that activate dendritic cells (DCs) and type 2 innate lymphoid cells (ILC2s), thereby promoting T helper (Th)2 differentiation and immunoglobulin E (IgE) class switching at the B cell level, a process highly dependent on IL-4 and IL-13 and leading to patient's sensitization upon IgE binding on their high-affinity Fc*ε*RI receptors on effector cells such as mast cells and basophils. Upon allergen re-exposure, IgE-mediated Fc*ε*RI crosslinking induces the rapid release of histamine, leukotrienes, prostaglandins, cytokines, and other inflammatory mediators, triggering the acute allergic response and sustaining tissue inflammation (3–5). Therefore, a hallmark of allergic disease pathogenesis is the overproduction of allergen-specific IgE. Beyond its immediate effector functions, IgE amplifies allergic inflammatory responses through its effects on antigen presentation, B cell activation, the recruitment of Th2 cells, and airway epithelial remodelling, thereby contributing to perpetuate the allergic cascade (6, 7). However, the contribution of IgE to antigen presentation and type 2 immune amplification is context dependent and varies according to the IgE receptor involved, with evidence supporting both pro-inflammatory and regulatory roles (8). Schroeder et al. showed that IgE neutralization with omalizumab markedly reduced Fc*ε*RI expression on pDCs and mDCs, which was associated with decreased DCs–dependent T-cell proliferation and reduced Th2 cytokine production (9). Hammad et al. also demonstrated that FcεRI-expressing inflammatory DCs are necessary and sufficient to initiate Th2 immunity *in vivo* (10). In addition, work by Eggel et al. demonstrates that FcεRI- and CD23-mediated IgE interactions might regulate distinct and complementary processes in different cell types: while FcεRI signalling pathways on effector cells mainly drive allergic effector responses, CD23 pathways on B cells/dendritic cells are also linked to IgE regulation and antigen presentation (8, 11).

The expanding understanding of IgE in allergic disease has provided a robust foundation for therapeutic interventions designed to modulate IgE–receptor interactions (12–14). Anti-IgE therapies, including omalizumab and other biologicals currently under development, such as ligelizumab among others, have demonstrated substantial clinical efficacy in allergic conditions by reducing free IgE levels and consequently downregulating FcεRI expression on mast cells, basophils, DCs and other cells (13). Although these therapies target key pathophysiological features of allergy, their mechanisms of action extend beyond IgE neutralization, encompassing broader immunomodulatory roles (14, 15).

**Figure 1:**
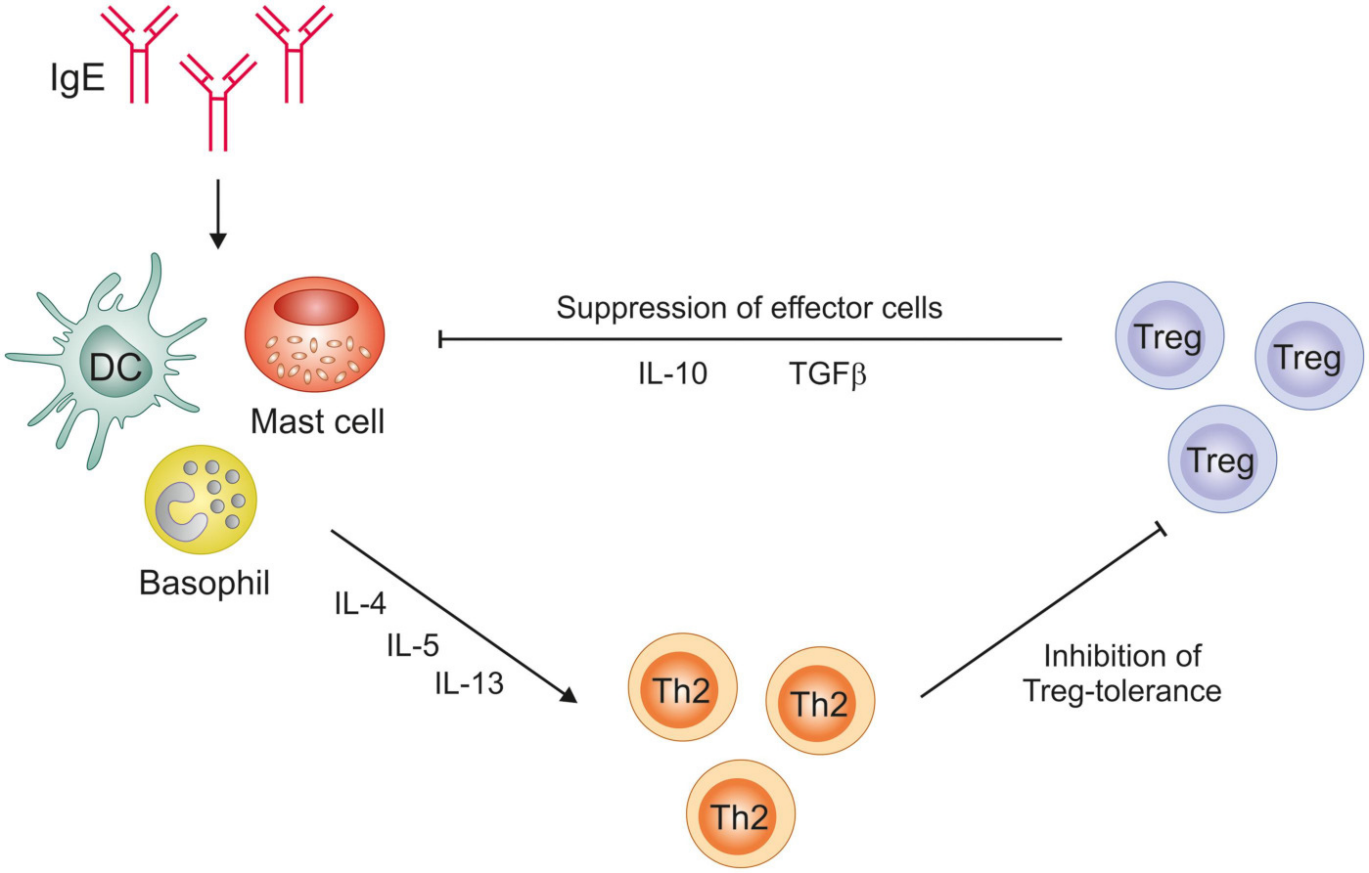
Network of IgE–effector cells–Tregs. IgE promotes mast cell, basophil, and proinflammatory DC activation, driving Th2 cytokine production and suppression of Treg differentiation. In turn, Tregs exert reciprocal inhibitory effects on these effector pathways by releasing IL-10 and TGF-β, thereby dampening allergic inflammation and promoting immune tolerance.

Regulatory T cells (Tregs) are pivotal regulators of immune tolerance in different settings and their functional alterations are associated with different diseases such as cancer, autoimmunity or allergic inflammation (15–18). IgE and Tregs engage in a functional interplay: IgE can impair Treg induction, while Tregs, in turn, regulate IgE production, forming a ke*y* axis in the control of allergic inflammation (Figure 1) (15, 19). The dynamic interplay between IgE-driven effector mechanisms and Treg-mediated suppression is still not fully delineated but is essential for defining new therapeutic paradigms.

This review aims to explore the interconnection between IgE and Tregs, examining how IgE levels and IgE-mediated signalling pathways may influence Treg generation and functional properties and, conversely, how Tregs modulate IgE production. We also discuss the potential of anti-IgE therapies not only to attenuate allergic inflammation but also to potentially restore immune tolerance by contributing to the regulation of Treg's function, which might well help to redefine their actual role in allergic inflammation.

## The role of IgE beyond mast cell activation

IgE, which exists predominantly in a receptor-bound state rather than as free antibody, is the least abundant antibody isotype in human serum. Nevertheless, IgE plays a main role in host defence against parasites, protection against venoms, immunoregulation and allergic inflammation (20–22). Structurally, soluble IgE is a monomer composed of two *ε* heavy chains and two light chains. The heavy chains contain four constant domains (Cε1–Cε4), with the Cε3 and Cε4 domains mediating the very high-affinity binding to its primary receptor, FcεRI (12).

Fc*ε*RI exists in two forms: a tetrameric αβγ_2_ complex on mast cells and basophils, and a trimeric αγ_2_ complex on DCs, plasmacytoid DCs (pDCs), monocytes, and eosinophils (23). The α chain binds IgE with sub-nanomolar affinity, while the β and γ chains contain immunoreceptor tyrosine-based activation motif (ITAM) motifs essential for intracellular signalling. IgE binding to FcεRI is essentially irreversible in physiological conditions, equipping these cells to rapidly respond to allergens. Although free IgE has a short plasma half-life (<1 day), receptor-bound IgE can persist on the cell surface for weeks, extending allergen sensitization (24). IgE also stabilizes Fc*ε*RI surface expression and enhances the survival of mast cells and basophils. This prolonged cellular persistence within tissues allows these cells to remain sensitized to allergens, leading to rapid and recurrent allergic responses upon re-exposure (25).

The low-affinity receptor CD23 (Fc*ε*RII) is a C-type lectin expressed mainly on B cells, as well as on myeloid and epithelial cells (12). It binds IgE independently of glycosylation and exists as both membrane-bound and soluble forms, with its oligomerization state modulating its affinity for IgE. Membrane CD23 regulates B-cell activation and facilitates the transport of IgE-antigen complexes to germinal centres, while soluble CD23 (produced by proteolytic cleavage) can enhance IgE synthesis and influence inflammatory signalling (11, 26).

IgE is classically associated with type I hypersensitivity reactions. Allergen mediated cross linking of Fc*ε*RI bound IgE on mast cells and basophils activates Src family and Tec family kinases such as SYK and Bruton's tyrosine kinase (BTK), leading to MAPK and NF-κB activation, degranulation, and release of histamine, tryptase, leukotrienes and cytokines that drive vasodilation, bronchoconstriction, mucus production and other hallmark allergic symptoms (27–30). In addition to mediating immediate hypersensitivity, BTK dependent signalling shapes the surrounding immune milieu by promoting production of pro-inflammatory cytokines such as IL-6, which can antagonize Treg differentiation and favour effector T cell responses (31). Beyond classical effector cells, IgE can modulate myeloid cell responses: IgE–Fc*ε*RI complexes on conventional DCs act as highly efficient endocytic receptors, so that allergen–IgE immune complexes are internalized, processed and presented on MHC-II to CD4^+^ T cells with 100–1,000 fold higher efficiency than antigen alone, thereby enhancing Th2 type responses (32, 33). Plasmacytoid DCs (pDCs) similarly engage IgE–FcεRI complexes, but in this context Fc*ε*RI signalling interferes with TLR7/9–IRF7 pathways and reduces type I interferon (IFN α) production, a defect that can be at least partially reversed by IgE neutralization with omalizumab (34, 35). Because type I IFNs promote tolerogenic DC programs, including up regulation of PD-L1, IDO and other regulatory molecules, IgE mediated suppression of IFN-α and concomitant increases in IL-6 may shift DCs away from a tolerogenic phenotype and impair Treg induction (36–38). Engagement of Fc*ε*RI on DCs and monocytes thus initiates intracellular signalling involving tyrosine phosphorylation, calcium mobilization and NF-κB activation that drives TNF-α, IL-6, IL-10 and chemokine production (e.g., CCL2, CCL28), while, through CD23 dependent pathways, monocytes further support B cell IgE class switching and reinforce the IgE–Th2 axis (33). By altering DC expression of IFN-α, PD-L1, IDO and IL-6, these IgE dependent pathways ultimately influence the transcriptional and epigenetic programs that govern FOXP3 expression and Treg lineage stability, including maintenance of the Treg specific demethylated region at the FOXP3 locus (39, 40).

As previously mentioned, a sustained elevation of IgE levels, whether due to atopy, chronic parasitic infection, or hyper-IgE syndromes, reshapes the immune microenvironment. High IgE levels, in addition to stabilizing Fc*ε*RI expression across mast cells, basophils, DCs, and monocytes, also reinforce type 2 cytokine networks, especially IL-4 and IL-13. This enhancement further promotes IgE class switching, strengthens Th2 polarization, and suppresses counter-regulatory pathways, thereby perpetuating inflammation in atopic diseases (3, 21, 41). Importantly, elevated IgE levels have also been observed in several non-allergic diseases, including certain forms of eczema, systemic lupus erythematosus, Crohn's disease (CD), and chronic urticaria. In these contexts, autoreactive IgE antibodies target self-antigens and activate mast cells or basophils independently of external allergens, sustaining inflammation and disrupting tolerance (15, 42).

## Regulatory T cells in allergic inflammation

Tregs are a specialized subset of CD4^+^ T cells with immunosuppressive functions that maintain immune homeostasis and peripheral tolerance, preventing the development of autoimmune and allergic disorders. Tregs are defined by the high expression of the IL-2 receptor α-chain (CD25), low or absent expression of the IL-7 receptor α-chain (CD127), and the presence of the transcription factor forkhead box P3 (FOXP3), which is indispensable for their development, function, and lineage stability (43, 44).

Tregs are divided into two major subpopulations: the natural Tregs (nTregs, also called thymic Tregs) and the induced Tregs (iTregs, also called peripheral Tregs). nTregs are generated and matured in the thymus upon self-antigen recognition (45–47), whereas iTregs derive from naïve CD4^+^ T cells in specialized peripheral tissues after the recognition of antigens from exogenous sources such as microbiota and bacterial products, dietary antigens, tumoral antigens, cannabinoids or allergens (48–53). Although the TCR repertoires of nTregs and iTregs are non-overlapping, they share similar immune suppressive mechanisms (46).

Tregs play a central role in the immunopathology of allergic diseases by restraining the proliferation and activation of effector T-helper subsets, including Th1, Th2, Th9, and Th17 cells. Tregs also participate in allergen-specific immune responses by limiting pro-inflammatory DCs and promoting the generation of tolerogenic DCs, thereby influencing effector T-cell priming. Moreover, Tregs modulate humoral immunity by suppressing allergen-specific IgE production while promoting the differentiation of IgG4-secreting B cells and IL-10-producing regulatory B cells. Additionally, Tregs regulate innate immune pathways by inhibiting the activation of ILC2s, natural killer T cells, mast cells, basophils, and eosinophils (54, 55).

Tregs exert their suppressive functions through various mechanisms, including direct cell-to-cell contact, secretion of inhibitory cytokines, cytolysis, and metabolic interference (56). Contact-dependent suppression involves the binding of cytotoxic T-lymphocyte antigen 4 (CTLA-4) to co-stimulatory molecules CD80/CD86 on DCs, leading to downregulation of these molecules. Additionally, Tregs remove antigen-MHC-II complexes from the surface of DCs via trans-endocytosis, thereby reducing antigen presentation (57). CTLA-4 engagement also promotes the upregulation of indoleamine 2,3-dioxygenase (IDO) in DCs, which depletes tryptophan essential for effector T cell proliferation (58). Other crucial inhibitory interactions include the binding of lymphocyte activation gene-3 (LAG-3) to MHC-II, which suppresses DC maturation, and the interaction between programmed cell death protein 1 (PD-1) and its ligand PD-L1 on T and B cells, resulting in the attenuated function of these lymphocytes (59). Furthermore, Tregs release immunoregulatory cytokines such as transforming growth factor-beta (TGF-β), interleukin-10 (IL-10), and interleukin-35 (IL-35), which inhibit the activation and proliferation of effector T and B cells and promote the induction of iTregs and regulatory B cells (Bregs) (60). Cytolytic activity mediated by perforin and granzyme pathways enables Tregs to induce apoptosis in target cells, including CD4^+^ and CD8^+^ effector T cells (61). Metabolically, Tregs express CD39 and CD73, which catalyze the conversion of extracellular ATP into adenosine, an immunosuppressive nucleoside that impairs DC antigen presentation and suppresses effector T cell proliferation via the adenosine A2A receptor (62). Additionally, constitutive expression of CD25 allows Tregs to deplete IL-2, limiting its availability and thereby constraining effector T cell expansion (63). These multifaceted mechanisms collectively enable Tregs to maintain immune homeostasis and prevent excessive or autoreactive immune responses.

In allergic diseases, allergen-specific Tregs play a key role in the generation and maintenance of immune tolerance to allergens (16, 17). Multiple studies support the concept that Treg destabilization or loss of suppressive function is a key contributor to allergic disease pathogenesis. In asthma patients, FOXP3^+^ Tregs exhibit reduced CCR5 expression, consistent with attenuated suppressive capacity (64). Tregs in asthma patients produce elevated IL-4 and display increased expression of chemoattractant receptor-homologous molecule expressed on Th2 cells (CRTH2), which is involved in disease exacerbation (65). In atopic dermatitis patients, it has been revealed a paradoxical increase in Tregs frequency linked with functional impairment, driven by dysregulation of the OX40/OX40L axis (66). Moreover, Tregs both in atopic dermatitis and food allergy exhibit Th2-like features, promoting disease pathogenesis (67, 68). Interestingly, Th2 reprogramming in Tregs impairs their tolerogenic function by downregulating TGF-β expression (69).

In addition to phenotypic instability, allergic patients often display quantitative Treg deficiencies. Individuals with severe asthma show reduced FOXP3^+^ Tregs in bronchoalveolar lavage and lower circulating Treg levels relative to healthy controls (70, 71). Similarly, several reports indicate decreased circulating FOXP3^+^ Tregs in allergic rhinitis (72). Patients with food allergy also exhibit reduced frequencies of circulating Tregs, including RORγt^+^ Tregs—an essential subset for maintaining tolerance to food and commensal antigens (73, 74). Interestingly, a recent phase 1 clinical trial using the Treg-selective IL-2 receptor agonist rezpegaldesleukin in atopic dermatitis demonstrated dose-dependent expansion of Tregs accompanied by robust and durable clinical improvement (75). Collectively, these findings demonstrate that disrupted Treg number, stability, and suppressive function are central mechanisms underlying the development and severity of allergic diseases.

In addition, T follicular regulatory (Tfr) cells represent a specialized subset of FOXP3^+^ Tregs mainly found in B cell follicles and germinal centers that regulate humoral immune responses (76, 77). In allergic disease, Tfr cells have been viewed as negative regulators of IgE production by limiting Tfh-driven B cell activation and class-switch recombination to IgE. Consistent with this suppressive role, patients with allergic rhinitis display unfunctional and reduced frequencies of circulating Tfr cells compared with healthy individuals (78, 79). However, recent murine studies have shown that activated Tfr cells may promote pathogenic IgE responses through IL-4 production and suppress protective IgG responses, leading to exacerbated allergic outcomes in specific contexts (80, 81).

## Anti-IgE therapy and regulatory T cells: emerging mechanisms

The expanding knowledge of the involvement of IgE and its receptors in the pathophysiology of allergic responses has established a robust foundation for the development of therapeutic strategies aimed at modulating IgE–receptor interactions (12). The pharmacological approaches targeting IgE were initially focused on neutralizing circulating free IgE, preventing activation of receptor-bound IgE, and inhibiting IgE production by targeting membrane-bound IgE in B cells (12, 82, 83). These principles led to the development of omalizumab, the first anti-IgE monoclonal antibody (mAb) for the treatment of allergic diseases. More recently, alternative anti-IgE biologicals have been generated and are currently undergoing evaluation in clinical trials at various stages, among which ligelizumab stands out as an anti-IgE mAb with higher affinity for free IgE than omalizumab (8, 13).

Omalizumab is approved for moderate-to-severe allergic asthma, chronic spontaneous urticaria (CSU), chronic rhinosinusitis with nasal polyps (CRSwNP) and for the treatment of IgE-mediated food allergy (12, 84). The mechanism of action of omalizumab involves masking the critical epitope required for free circulating IgE to bind to the high-affinity Fc*ε*RI and low affinity CD23 receptors. By inhibiting the interaction of IgE with FcεRI on mast cells and basophils, the pool of receptor-bound IgE on these effector cells is progressively reduced, thereby limiting the capacity for IgE-mediated cross-linking and subsequent degranulation upon allergen exposure. Furthermore, omalizumab induces downregulation of Fc*ε*RI expression on effector cells, which further decreases the availability of receptor sites for unbound circulating IgE (14). Comparative structural analyses have demonstrated that ligelizumab and omalizumab differ in their capacity to inhibit IgE binding to the high- and low-affinity receptors (FcεRI and CD23, respectively), reflecting their differential epitope recognition on IgE and their varying capacities to induce conformational changes upon binding. These molecular differences may underlie the diverse clinical outcomes observed with these monoclonal antibodies across different IgE-mediated diseases (8, 85).

**Figure 2:**
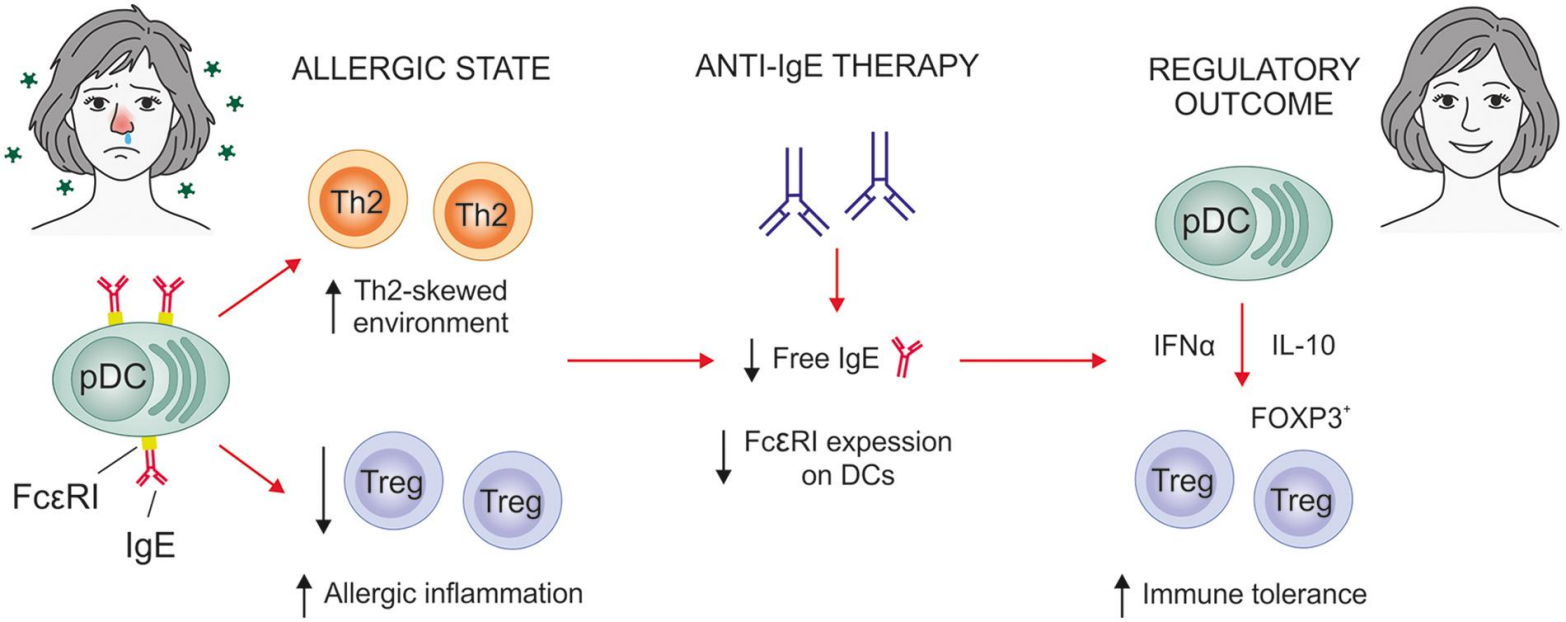
Mechanistic model of anti-IgE effects on Tregs. The cross-linking of IgE with its high affinity receptor FcεRI in pDCs induces allergic inflammation by promoting a Th2-skewed environment and decreasing Treg generation. Anti-IgE therapy reduces free IgE levels and FcεRI expression on DCs, promoting a shift toward a tolerogenic phenotype that favours Treg induction. The expansion of Tregs contributes to restore immune balance and clinical improvement in allergic patients.

A bidirectional relationship has been described between IgE and Tregs. On one hand, a deficiency in Tregs leads to elevated serum immunoglobulin levels, including increased concentrations of autoantibodies and IgE (15, 19). However, a recent clinical trial using low-dose IL-2 to selectively expand Tregs in allergic patients has also demonstrated clinical improvement, despite the absence of detectable changes in allergen-specific IgE or IgG levels (86). On the other hand, in human pDCs derived from atopic donors, IgE–Fc*ε*RI cross-linking has been shown to impair their capacity to promote Treg induction *in vitro* (87), suggesting that IgE itself can negatively influence Treg differentiation and function (Figure 2). Moreover, different studies have explored how T follicular regulatory (T_FR_) cells modulate IgE responses, further supporting the dynamic interplay between IgE regulation and Treg-mediated immune tolerance. Clement RL et al. demonstrated that CXCR5^+^ Tregs regulate both autoreactive and allergen-specific IgE^+^ B cells, thereby controlling IgG and IgE responses to vaccines, allergens, and autoantigens, and exerting essential immunoregulatory functions (88). In murine models, T_FR_ cells exhibit a suppressive effect on IgE responses in allergic airway disease. In humans, T_FR_ cells are also associated with reduced allergic responses, although direct *in vivo* evidence of their suppressive effect on IgE remains limited (81).

In children with asthma, omalizumab treatment has demonstrated an increase in Treg frequency, which is associated with better asthma control (89). Different studies have studied the mechanisms by which omalizumab can influence Tregs generation. It has been demonstrated that IgE-FcεRI crosslinking in Toll-like receptor 9 (TLR9) ligand-activated pDCs impairs the generation of FOXP3^+^ Tregs and favours Th2 allergic profiles, which is restored by pre-treatment of pDCs with omalizumab (Figure 2) (87). The function of pDCs has been reported to be altered in asthma patients, and reduced pDCs levels during childhood are associated with an increased risk of developing asthma (90–92). These cells express high levels of FcεRI and therefore, they play a crucial role in the restoration of Tregs generation by omalizumab. Similarly, the capacity of the anti-IgE mAb ligelizumab to regulate the functional properties of pDCs has been also studied (93). Due to the higher affinity for free IgE of ligelizumab, it blocks the binding of free IgE to Fc*ε*RI on pDCs more efficiently than omalizumab and can restore the capacity of these cells to generate FOXP3^+^ Tregs and to produce high levels of IFN-α (93). This novel molecular mechanism underlying the mode of action of different anti-IgE mAb has also been explored in other inflammatory disorders involving high levels of IgE and alterations in the number of Tregs such as in CD (94). CD is an inflammatory bowel disease involving recurrent, destructive pathological inflammation that leads to progressive gut damage (95). A growing body of research has investigated the possible association between CD and asthma, and substantial recent evidence suggests a causal connection between them (96, 97). It has been demonstrated that IgE could impair Treg function in CD patients with a potential involvement of IgE-pDCs-Tregs axis in the regulation of inflammatory responses in CD. In this context, omalizumab has also shown capacity to restore Treg generation from CD patients, strengthening its potential as a modulator of of Tregs homeostasis in inflammatory diseases characterized by high levels of serum IgE (94).

## Clinical implications and future perspectives

Compiled experimental evidence with anti-IgE biologicals such as omalizumab has demonstrated substantial efficacy in reducing allergic inflammation and symptom burden in different IgE-mediated disorders (7, 98, 99). However, as mentioned before, accumulating evidence suggests that IgE modulation may exert broader immunoregulatory effects than initially expected, particularly on Treg populations (87, 93). This observation raises the intriguing possibility that anti-IgE therapy might not only suppress effector pathways but also help restore immune tolerance. Nevertheless, whether these effects are transient or can induce long-lasting immune reprogramming remains an open question.

Beyond anti-IgE therapies, similar immunomodulatory patterns are emerging with other biologics used in allergic diseases that do not directly target IgE. Recent studies indicate that agents blocking Th2 cytokine pathways, such as anti–IL-4Rα or anti–IL-5/IL-5R mAbs, can also promote Treg expansion and activity. For instance, a 6-month treatment with dupilumab, an anti-IL-4Rα mAb, in patients with atopic dermatitis not only reduced Th2 cell populations but also enhanced Treg activity, particularly among IL-10-producing Tregs (100). Likewise, in a cohort of severe asthma patients treated with anti-IL-5/IL-5R mAbs (mepolizumab and benralizumab) for 24 months, an increase in naïve T cells, terminally differentiated effector memory (TEMRA) cells, and Tregs was observed (101), consistent with previous findings in severe eosinophilic asthma (102). Together, these findings suggest that biologic therapies targeting Th2 cytokine pathways may exert broader immunomodulatory effects, potentially restoring immune balance through the expansion of Treg subsets, a mechanism already well established for allergen-specific immunotherapy (103, 104).

In parallel, therapeutic strategies aimed explicitly at boosting Treg number or function represent an exciting avenue for long-term disease control (15, 105). Enhancing Treg activity could provide a route toward sustained tolerance rather than temporary suppression of symptoms (16). Moreover, Tregs themselves have potential as biomarkers of therapeutic response, offering a measurable correlation of immune homeostasis restoration.

## Conclusions

The growing body of evidence linking IgE biology with Treg activity has reshaped our understanding of the mechanisms underlying allergic diseases beyond the classical effector paradigm. IgE not only drives mast cell and basophil activation but also influences the functional properties of DCs and, consequently, the fine balance between effector and regulatory immune responses. Anti-IgE therapies, initially conceived to neutralize circulating free IgE and prevent IgE-Fc*ε*RI-mediated activation of effector cells, are now recognized to exert broader immunomodulatory effects. By reducing free IgE levels and downregulating Fc*ε*RI expression on DCs, these treatments can promote a tolerogenic phenotype that enhances Treg induction and function.

This emerging perspective highlights that the clinical efficacy of anti-IgE treatments may, at least in part, derive also from their ability to reestablish immune tolerance rather than by solely suppressing allergic inflammation. Thus, therapeutic success in allergy management should be viewed not only in terms of symptom control but also as a potential step toward immune reprogramming. Elucidating the molecular mechanisms through which anti-IgE and other biologicals influence specific immune cell populations in IgE-mediated disorders might provide valuable insights for interpreting clinical outcomes and optimizing the design of future clinical trials.

Overall, anti-IgE therapy is broadly effective because it is not allergen-specific, but this same feature might limit its long-term durability. Treatments capable of expanding or stabilizing Tregs may ultimately achieve the long-sought goal of durable disease modification. Understanding and harnessing the potential shared mechanisms of tolerance induction across anti-IgE therapy, biologicals targeting specific cytokines or their receptors, and allergen-specific immunotherapy will be key to developing next-generation interventions aimed at restoring lasting immune balance.
